# Roadmap for creating an accelerated three-year medical education program

**DOI:** 10.1080/10872981.2017.1396172

**Published:** 2017-11-08

**Authors:** Shou Ling Leong, Joan Cangiarella, Tonya Fancher, Lisa Dodson, Colleen Grochowski, Vicky Harnik, Carol Hustedde, Betsy Jones, Christina Kelly, Allison Macerollo, Annette C. Reboli, Melvin Rosenfeld, Kristen Rundell, Tina Thompson, Robert Whyte, Martin Pusic

**Affiliations:** ^a^ Department of Family and Community Medicine, Penn State College of Medicine, Hershey, PA, USA; ^b^ Department of Pathology, NYU School of Medicine, New York, NY, USA; ^c^ Davis, School of Medicine, Office of Medical Education, University of California, Sacramento, CA, USA; ^d^ Department of Family and Community Medicine, Medical College of Wisconsin, Wausau, WI, USA; ^e^ Office of Curricular Affairs, Duke University School of Medicine, Durham, NC, USA; ^f^ Department of Family and Community Medicine, University of Kentucky College of Medicine, Lexington, KY, USA; ^g^ Departments of Medical Education and Family Medicine, Texas Tech University Health Sciences Center School of Medicine, Lubbock, TX, USA; ^h^ Department of Family Medicine, Memorial Health Family Medicine Residency Program, Savannah, GA, USA; ^i^ Department of Family Medicine, Family Medicine at Care Point East, Ohio State University, Columbus, OH, USA; ^j^ Department of Medicine, Cooper Medical School of Rowan University, Camden, NJ, USA; ^k^ Department of Family Medicine, The Ohio State University, Columbus, OH, USA; ^l^ Office of Medical Education, Mercer University School of Medicine, Savannah, GA, USA; ^m^ Office of Undergraduate Medical Education, McMaster University, Hamilton, ON, Canada; ^n^ NYU School of Medicine, Institute for Innovations in Medical Education, New York, NY, USA.

**Keywords:** Medical education, innovation in medical education, accelerated pathways, accelerated medical degree

## Abstract

Medical education is undergoing significant transformation. Many medical schools are moving away from the concept of seat time to competency-based education and introducing flexibility in the curriculum that allows individualization. In response to rising student debt and the anticipated physician shortage, 35% of US medical schools are considering the development of accelerated pathways. The roadmap described in this paper is grounded in the experiences of the Consortium of Accelerated Medical Pathway Programs (CAMPP) members in the development, implementation, and evaluation of one type of accelerated pathway: the three-year MD program. Strategies include developing a mission that guides curricular development – meeting regulatory requirements, attaining institutional buy-in and resources necessary to support the programs, including student assessment and mentoring – and program evaluation. Accelerated programs offer opportunities to innovate and integrate a mission benefitting students and the public.

**Abbreviations**: CAMPP: Consortium of accelerated medical pathway programs; GME: Graduate medical education; LCME: Liaison committee on medical education; NRMP: National residency matching program; UME: Undergraduate medical education

## Background

Medical education is evolving as a large number of colleges of medicine are currently undergoing substantial curricular changes. Although the traditional framework of two-year didactic and two-year clinical educational has been stable since its adoption after the Flexner Report [1].

**Figure 1.**
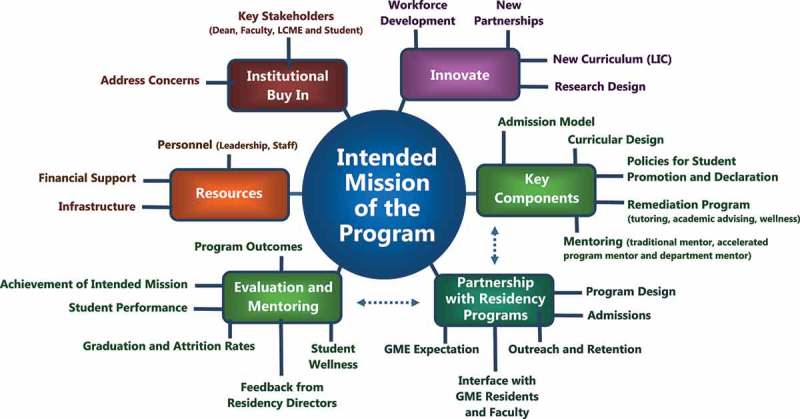
Creating an accelerated three-year medical education program.

The Institute of Medicine, Association of American Medical Colleges (AAMC), American Medical Association, and the Josiah Macy Jr. Foundation have all called for education reform to better align medical training with 21st century healthcare needs [2]. The 2010 Carnegie Report recommended competency-based and individualized education, including the option of fast tracking medical education. Rising student debt and the expectation for a shortage of physicians have reinvigorated interest in three-year accelerated MD programs [3]. In 2014, 35% of US medical school deans reported interest in developing an accelerated program [4].

With support from the Josiah Macy Jr. Foundation, eight medical schools (now 12) with three-year medical pathway programs founded the Consortium of Accelerated Medical Pathway Programs (CAMPP). While the CAMPP pathways vary in size, specialty focus, mission, and curricular elements [5], the consortium shares best practices and conducts collaborative studies on this innovative model of training. This paper provides guidance to institutions considering the implementation of three-year medical pathway programs based on a review of the current literature and the collective experiences of the CAMPP medical schools during the development, implementation, and evaluation of their programs, Table 1.

**Table 1.** Best practices for implementing a three-year accelerated program from the Consortium of Accelerated Medical Pathway Programs (CAMPP) schools

Steps in creating a three-year program	Mission	Admission model	Curricular plan	Policies for advancement and deceleration	Remediation program	Mentor program	Partnerships with residency programs	Program evaluation	Ensure sufficient resources	Get buy-in and address concerns	Innovate
**Best practices**	Address shortage of primary care physicians Schools: 1, 4, 5, 7, 8, 9, 10	Admission at matriculation Schools: 1, 3, 4, 5, 6, 7, 8, 9, 10	Summer classes School: 1, 2, 5, 6, 7, 8, 9, 10	Must meet school policy for advancement; Must pass all courses and clerkships and Step 1 and 2 School: all	Establish policies for remediation to maintain program excellence while providing a fair chance for the students to succeed School: all	Each student assigned a traditional advisor, an accelerated pathway advisor, and a departmental advisor School: 6, 7, 10	Conditional acceptance to affiliated residency program School: 1, 2, 5, 6, 7, 8, 9, 10	Student performance (exam scores, narratives, course and clerkship grades, Step scores) is tracked. School: 1, 6, 7, 8, 10	Institutional leadership and financial support School: all	Obtain approval from key institutional stakeholders (Dean, Curriculum Committee, Educational Affairs, residency program directors and finance office) and LCME School: all	Longitudinal integrated clerkship School: 1, 4, 7, 9, 10
	Address workforce shortage of the state (psychiatry, general surgery, primary care and underserved areas) Schools: 4, 5, 8, 9, 10	Delayed admission – year 1 of medical school School: 5, 7	Additional courses alongside traditional curriculum School: 7, 8, 9, 10	Deceleration addressed in grading, promotions, and appeals policy School: 1, 6, 7, 8, 9, 10	Decelerate to four-year curriculum for significant academic issue or specialty choice areas. Remain on regional campus after deceleration School: 4, 10	Faculty navigators serve as advisors. Need to connect to main campus for specialty academic advising School: 4	Students can apply to any residency programs School: 3, 4	Student performance, specific student survey, and residency program director surveys School: 2, 6, 7, 8, 10	HRSA and external support School: 1, 7, 8, 9, 10	Invite faculty from successfully launched accelerated programs to your institution School: 1	Distance learning, with digital classroom School: 4
	Reduce student debt Schools: 1, 2, 4, 5, 6, 7, 8, 9, 10	Dual paths- at matriculation and at year 1 of medical school Schools: 5, 6,7, 8,	Problem-based learning School: 3, 5, Case-based learning school: 4	Final determination about advancement or deceleration made in December of third year School: 2		In addition to traditional mentoring program, students are assigned a primary care physician coach. School: 9	UME-GME continuum of training and tracking School: 1, 2, 5, 6, 7, 8, 9	Research assessment pilot using Wise-Oncall and simulation School: 6	Philanthropy, community support for regional campus School: 4		Patient-centered medical home curriculum School: 1, 7, 10
	Individualized pathway Schools: 6, 7	Community engaged admission process School: 4	Early clinical exposure to patients School: 3, 5, 7, 8, 9, 10			Each student has a faculty mentor and a resident mentor for their primary care specialty School: 1	Residency program director is part of the Accelerated Pathway team and takes part in the interviews School: 1, 2, 5, 6, 7, 8, 9, 10	OSCE and simulation evaluation School: 4, 7, 10	State funding for program that allows tuition scholarships School: 5, 10		
	Improve educational efficiency toward orthopaedics residency School: 2, 7	Admission to the entire class School: 3	Curriculum on community engagement, population health School: 4, 5, 10			Mentor includes Dean in medical school, Vice-chair of department, faculty member, resident and graduates of the program School: 2	Opportunities for students to be integrated into the GME programs School: 2, 6, 7, 8, 9	Tracking performance in UME and GME. School: all	Supported by the Department of Orthopaedics School: 2		
	Consider relevance of clinical experience / patient experience toward career competencies School: 2	Admission after preclinical and half of the clinical year School: 2	Competency-based education School: 7, 8, 9, 10					Wellness tracked through regular meetings with students. School: 1, 6, 7, 8			
	Reprioritize opportunities for meaningful research School: 2		Primary care focused, team-based care School: 7, 9								

## Align the accelerated pathway with the intended mission

This paper provides recommendations for medical schools that wish to or are establishing accelerated curricula. An accelerated program requires a clear understanding of what it intends to accomplish, the mechanisms to be employed, and the benefits of the change. Importantly, key stakeholders should create the mission statement before the program is established so that the mission serves as the guide to program development. The mission is central to all components of the program including the development of the curricular design, admission policy, allocation support, and allocation of resources, Figure 1.

The mission for accelerated pathways can vary but often includes intending to move from a time-based to a competency-based education model [1]. Other missions include developing a workforce plan that improves physician geographic distribution through community engagement and collaboration [6]; addressing physician specialty shortages such as primary care and psychiatry; targeting/streamlining training for subspecialties [7]; and focusing on care for specific populations such as Native American or inner city urban poor [8]. The mission may also specifically target the cost of medical training by reducing the overall tuition [3].

## Develop the admission model

The mission of the program should guide the recruitment, application, interview, and selection processes. A mission to educate family medicine physicians to serve rural communities will require a different admissions model compared with one designed to educate surgical subspecialists. Those programs that offer a conditional acceptance to residency programs (see below) will need to develop a process that includes Graduate Medical Education (GME) stakeholders in the decision making.

There are two primary models for student admission: direct admission at matriculation or delayed admission, usually within the first months of medical school [5,7]. These models offer unique advantages and disadvantages to both the applicant and the institution.

With the direct admission model, candidates can be specifically targeted with this method, particularly non-traditional second career or those with a clear pre-matriculation affinity for a given specialty. However, identifying reliable predictive factors can be difficult. More holistic information such as work and life history and a demonstrated commitment to the specialty should supplement the conventional dataset to provide a complete picture of the ideal candidate. Behavioral interviewing involving both the Undergraduate Medical Education (UME) and where relevant, GME stakeholders is necessary to assess non-quantitative qualities (maturity, resilience, motivation, program fit) that will impact student and program success [9,10]. The direct admission process offers an excellent recruitment opportunity, especially for a combined UME-GME program.

The delayed admission model provides for the selection of students during the first year of medical school. This model allows for the review of student performance in the medical education program prior to accepting the applicant into the accelerated curriculum, increasing the likelihood that the student can successfully navigate the academic rigor of the accelerated program. In this model, prospective applicants are provided opportunities to interact with the program faculty to assess student/program fit. Many schools offer early clinical experiences allowing the candidate to more substantively assess the career match. Accelerated programs can use a combination of these two admission models to optimize student recruitment, retention, and success.

## Develop and implement key components of the pathway

### Construct a curricular plan

Two core program objectives among Liaison Committee on Medical Education (LCME) accredited medical schools are particularly relevant to three-year pathways: a curriculum with ‘sufficient breadth and depth to prepare medical students for any residency program and subsequent contemporary practice of medicine,’ and an education program that includes at least 130 weeks of instruction [11]. It is helpful to address the practical issues of curriculum development to determine content, timing, and pacing of courses and clerkships. For institutions with both accelerated and non-accelerated tracks, a careful review of the instruction calendar may identify available time (e.g., during pre-matriculation, summers, or fourth year) or areas of redundancy in the curriculum that can be made more efficient during the pre-clinical or clinical training phases. Several of the CAMPP schools utilize the summer between year one and two of medical school to add coursework for the accelerated track. Other schools added clinical courses alongside the school’s traditional curriculum.

Accelerated pathway students should clearly and transparently meet all graduation requirements. Each institution’s governance structure has oversight to ensure compliance with graduation requirements that for all students. As with a traditional curriculum, schools need to develop explicit moments in the curriculum when determinations are made regarding student advancement. These typically occur at the end of units of curriculum or academic years but will need to be accelerated to appropriate points in the three-year or other accelerated cycle [12].

Understanding that development will proceed at different rates for different students, programs must define the minimum pace of advancement students must achieve to progress as on track. For some CAMPP schools, a failure in a clerkship exam would necessitate the student to decelerate back to the four-year program. Timing of assessments may need to be adjusted to take into account the different timing of promotion/advancement decisions [13]. The perception of assessment by both students and faculty should also be monitored. Some could feel extra assessment pressure in an accelerated program. Differential assessment and standards in the same institution can also be sensitive issues [14].

The shortened curriculum in US schools must continue to ensure that students are adequately prepared for licensing examinations. Programs must also provide opportunities to inculcate and assess Core Entrustable Professional Activities for Entering Residency, particularly in clinical clerkship training [15–17]. For students who need remediation, have life events, or change their minds, provision needs to be made for enough comparability, in terms of course structure and curricular content, so that they can move into a non-accelerated track.

The school’s core program objectives determine what cannot be lost to acceleration. Most accelerated pathway institutions have removed curricular content related to the fourth year, e.g., away rotations, audition rotations, interview time, vacation, etc. Other schools have developed longitudinal integrated clerkships to reduce redundancies in clinical training, and others have created experiences that students choose in place of or concurrent with the non-accelerated curriculum.

### Develop policies and practices for determining student promotion and deceleration

While accelerated students will meet the same graduation requirements as the four-year pathway students, promotion decisions may differ [11]. Likely, most of the promotions criteria will be the similar to the four-year program either on an accelerated timeframe or with some modifications. Some criteria may be added or adjusted in order to accomplish the mission-specific objectives of the accelerated program. Consideration will need to be given as to whether the same promotions committee that adjudicates the four-year program can serve for the three-year or if there is a need to form a separate committee.

Unique to accelerated pathways is the option of ‘deceleration’ to the four-year pathway. This requires curricular and assessment comparability which may not be possible after certain milestones have passed. Any number of academic or professional reasons could lead to deceleration including a preference by the student to decelerate; physical or mental health issues; pregnancy and parental leave, or career indecision requiring more exploration time [18,19].

Policies and practices need to govern if, when, and how students can leave the accelerated program. For students who are not succeeding in the accelerated program, careful promotions consideration will determine whether the student simply ‘decelerates’ to the four-year pathway or requires customized remediation. There may be a tendency where schools run concurrent three- and four-year curricula to use the four-year program as the back-up when students require remediation. It is important for schools to explicitly determine whether this is the best approach for all scenarios, for some pre-determined situations or whether every situation will be examined on a case-by-case basis. Where the three-year program includes a return of tuition savings, decisions about tuition repayment need to be included in the policies.

*Due to the compact nature of an accelerated program, there is limited flexibility for remediation. Student who fails a clerkship exam will likely need to decelerate back to the four-year program. With repeated academic difficulties, student in a four-year program may need additional time to graduate.*

*A s* *tudent who decides a different career path than the intended mission of the accelerated program may decelerate back to the traditional four-year program, which allows students to apply to any residency programs. On some occasions when a school offers multiple accelerated programs in various specialties, a student may request to transfer from one specialty program to another. For example, a student enrolled in a pediatric accelerated program may request to transfer to the medicine accelerated program within the same school, if space is available. If the student received a scholarship for the accelerated program, deceleration or transfer may cause the scholarship to revert to a loan, depending on the school’s policy.*

### Develop a customized remediation program for students in an accelerated pathway

Due to the shortened nature of an accelerated pathway program, students who require remediation may not be able to repeat a portion of the curriculum or have the additional time needed to remediate. The tight timeline for the licensing examinations may require transition out of an accelerated pathway, as described above. Each accelerated program must match the structure of the review process to the needs of the student and the resources available for remediation. A process to determine the needs of the student should be outlined to understand if the remediation needed is in the area of medical knowledge, skills, attitudes, behaviour, or professionalism. Resources available for remediation likely need to include tutoring, counseling, wellness, and academic advising [20,21].

The remediation review process and whether it will function independently from the standard remediation process must be defined. Using the existing process has the advantage of established protocol and experienced counsellors, but the disadvantage of no prior precedent for an accelerated student. If a new committee is to be organized, key additional members should include an Academic Program Director with experience in the accelerated track curriculum and/or a program mentor as an advocate.

### Create a mentoring program

Mentoring is a key component of an accelerated program, especially given its increased intensity. Most programs use a group of faculty and peer mentors who focus on complementary aspects of a student’s experience [22]. For example, faculty mentoring at NYU includes three mentors: a traditional mentor, an accelerated program mentor, and a departmental mentor. The traditional mentor is an academic coach who follows a student throughout their medical school training. An accelerated pathway advisor understands the curriculum, logistics, stressors, and difficulties that may be unique to an accelerated student. This advisor gives significant attention to professional development and maturation and to student wellness, and meets with the students several times a year. In programs with direct transition to residency, a departmental advisor is assigned from the prospective residency program. This advisor is responsible for acclimating the student to the department by identifying appropriate departmental activities (such as grand rounds, shadowing opportunities, social events, research experiences) for the student to attend. Mentoring that includes early engagement by faculty and residents from the chosen residency program provides students with enough exposure to their field of choice to solidify their residency decision, or if necessary, change their residency and transition out of the program. Near-peer mentoring by more senior medical students is a promising innovation for accelerated pathway students just as it is for the regular pathway.

## Develop meaningful partnerships with target residency programs

GME programs and residency program directors are key partners with accelerated UME programs [23]. To the extent possible, program directors should be involved in all aspects of the UME program including program design and implementation, admissions, outreach and retention programs, and faculty selection and development. Early and frequent interface between students and GME faculty and residents helps to build the partnerships and helps students easily develop a sense of belonging in the GME space [24].

Many accelerated UME programs offer a conditional acceptance to a partner GME program. Conditions of acceptance into GME are defined by UME and GME partners and often include academic and clinical performance expectations. Both UME and GME programs need to agree on the holistic attributes that ensure, at the time of interview for medical school, a medical student will be a good GME fit. Residency program stakeholders should contribute to advancement and deceleration decisions. Most programs build-in opportunities for GME programs to work with students during medical school.

Knowing the GME program expectations of their day-one interns is helpful in designing the post-clerkship experiences for medical students, potentially easing the transition. In many of the accelerated UME programs, the post-clerkship curriculum is tailored to the students’ target GME field. GME programs must comply with National Residency Matching Program (NRMP) [25] guidelines and most accelerated pathway programs with partner residency programs participate in the match [25]. All-in policy exceptions to the NRMP match have been granted for the Rural Scholars Programs and the Family Medicine Accelerated Programs.

## Plan for program evaluation and monitoring

After the regulatory requirements have been met, it is important to consider program evaluation methods in order to assess its effectiveness in achieving stated outcomes [26]. Just as with assessment, program evaluation should employ multiple methods for systematically studying program outcomes. Various evaluation methods should be considered including objective-oriented, process-oriented, and participant-oriented [27].

Accelerated pathways typically use the same base program evaluation metrics that are used in the traditional curriculum [28], including student performance on local and standardized exams, student advancement, graduation, and attrition rates (the latter being of particular importance for accelerated programs), NRMP match rates, and surveys of pathway graduates and their program directors. Tracking and comparing these objective-oriented data for students in the traditional curriculum and the accelerated pathway may highlight the comparable strengths and weaknesses of each.

As schools implement accelerated pathways with defined missions and associated goals, program evaluation elements should assess the achievement of these goals. Additional elements could include pathway-specific open-ended questions on course evaluations and surveys of the pathways’ graduates and their program directors. Focus groups are beneficial and may be designed to include sessions with students selected into the accelerated pathway, students who applied but were not accepted into the pathway (if applicable), and students who chose the four-year curriculum over the accelerated pathway. This would allow for comparison of student perceptions (participant-oriented) between four-year and accelerated pathway students.

Additional information about students’ experience in an accelerated pathway and the intended goals of the pathway may be gleaned through surveys measuring student characteristics like those now included on the AAMC Graduation Questionnaire – surveys assessing grit, burnout, and empathy for example. Comparing accelerated pathway students’ responses to those of students in the traditional curriculum may highlight the impact of each on students and signal if an adjustment is required.

## Ensure that sufficient resources (personnel, infrastructure, and money) are in place to effectively run an accelerated pathway

From a personnel standpoint, at a minimum all programs should identify a lead faculty or dean level individual to oversee the program and a faculty advisor for the students. A lead faculty member should dedicate at least 0.25 full time equivalent (FTE), depending on the size of the program and the number of residency programs involved. An administrative assistant to manage logistics, scheduling complexities, and non-traditional deadlines is required for bigger programs; while a smaller, less complex program may get by with less support. Programs that include multiple residency partnerships should identify department specific faculty mentors to ensure that their students are well integrated into the department [7].

Some CAMPP programs experienced an increase in the number and complexity of applications necessitating additional staffing. Others were able to use existing resources and bring the undergraduate and residency program admissions teams together. Additional personnel may be needed to sit on a new Executive or Promotions Committees.

It will be necessary to consider whether your program will have any additional curricular programming or assessments specific for the accelerated pathway. For example, additional assessments for remediation or promotion, or additional curricular content (i.e., pre-matriculation courses to ensure compliance with the LCME’s 130 weeks of curriculum) are likely associated with additional cost.

## Get buy-in and address concerns

Buy-in from key stakeholders and institutional decision makers is crucial to the success of novel educational programs that can effect a paradigm shift. Key stakeholders include: the Dean, the Curriculum Committee and associated Subcommittees, the Office of Academic Affairs or Medical Education, the Residency Program Directors, the faculty, and the institution’s Finance Office. The LCME is another key early stakeholder. Standard 6 of the LCME standards requires that the faculty of the medical school be responsible for the detailed design and implementation of the components of the curriculum. The LCME’s Element 8.1 addresses curriculum management and the central role of the Curriculum Committee (a Faculty Committee) in the oversight of the medical education program [11]. The Dean, as Chief Academic Officer, has ultimate responsibility for educational programs and controls school resources. The support of the Dean is essential. There are key steps that should be taken to build support and address concerns for an accelerated pathway program. These include the development of a detailed plan for the program before you approach stakeholders and obtaining the early support of the senior leadership. Understanding the opinion of key leaders and engaging major stakeholders will allow them to participate in program development. Students are also important stakeholders. Their feedback should be sought, especially with the curricular development. Inviting faculty from successfully launched accelerated programs to your institution will help address stakeholders’ concerns. The LCME requires notification of the creation of new parallel curriculum. The LCME website has a white paper defining a parallel curriculum and a notification form that must be submitted one year before the expected date of implementation [11].

A limitation of this manuscript is that, with the exception of McMaster University, which offers a three-year program to all students, the CAMPP schools offer accelerated program to relatively small cohorts of students (range 2 to 50). As such, one should extrapolate these recommendations to large, single cohorts with caution. In particular, one would lose the built-in natural experiment that accrues when both tracks are present in a medical school.

In the Medical College of Wisconsin, the regional campuses at Central Wisconsin and the Green Bay offer a three-year accelerated program to 50 students combined.

Larger cohort size of students normalizes the accelerated track to students, faculty, and external stakeholders and can reduce the isolation of students in the accelerated track. The school can create standard messaging and processes for all students. A regional campus that is away from the main campus, allows some institutional flexibility to adjust to the size of the cohort.

## Innovate!

Most accelerated programs are generally viewed as a school’s innovative initiative that is designed de novo. Creation requires buy-in from key leadership of the institution. In this setting, accelerated programs have special opportunities to incorporate novel features in the education program.

Accelerated pathways can illuminate the social contract of medical schools with the society at large. Several schools in the CAMPP (Penn State, Medical College of Wisconsin, Mercer, Texas Tech, UC Davis) are using accelerated pathways as a framework to align training with the healthcare needs locally and nationally [2]. With the social mission to address physician shortages, these accelerated programs were designed to increase the number and accelerate the entry of medical graduates in shortage areas, such as in primary care, general surgery, and psychiatry specialties; and in rural and underserved communities.

Schools have created innovative new partnerships to develop their programs [29]. With the shared mission to produce more primary care graduates who are well prepared to serve the health needs of California’s diverse population, UC Davis School of Medicine and Kaiser Permanente Northern California formed a partnership in the development and implementation of the UC Davis accelerated program.

To enhance curricular efficiency and continuity, some schools are using longitudinal integrated clerkship (LIC) instead of the traditional block clerkships where the students complete all the clerkships longitudinally. LIC students follow a panel of patients during the clinical year, creating opportunities to foster meaningful relationships with patients and preceptors [30,31].

In many schools, acceleration has created a ‘natural experiment’ where two different curricular models run side-by-side. The accelerated pathway can benefit teaching and learning in the standard pathway by providing a contrast based on longitudinal time scale. For example, the benefits of fourth year electives can be more carefully delineated when there is a natural control group who proceeded to residency without them.

Similarly, the extent to which individualized education can be competency-based can be assessed for a cohort of learners with early interest and commitment to a specialty. Other topics of innovation could include economic investigations as to the degree to which student debt burden is reduced and consideration of the effect on professional identity formation of the enhanced mentorship and support of the GME faculty.

## Summary

In general, the structure and processes necessary to establish a three-year MD accelerated pathway are similar to those required of a standard medical school, with some added complexities that require both careful planning and additional resources. The timing and nature of assessments need to accurately reflect the student’s progress but be provided in time for specific acceleration/deceleration decisions as well as being substrate for program level evaluation of the accelerated pathway. Successful programs go beyond simply compressing existing programs but rather use this organizational innovation to re-engineer existing processes so as to better align and integrate the program in service of a well-defined mission, benefitting both the student and the public.
